# Unraveling the personality traits of civil heroes in great disaster: a qualitative study

**DOI:** 10.3389/fpsyg.2025.1512948

**Published:** 2025-03-24

**Authors:** Haili Hu, Dan Ou

**Affiliations:** College of Marxism, Southwest Jiaotong University, Chengdu, China

**Keywords:** civil heroes, personality traits, disaster, qualitative study, thematic analysis, semi-structured interviews

## Abstract

**Introduction:**

Heroes are everywhere, and shine the brightest in the dark. Although the concept of heroism has been widely discussed in the fields of philosophy and social sciences, the personality traits of civil heroes emerging from great disasters has remained unexplored topic.

**Methods:**

The qualitative research methodologies are conducted to dissect and elucidate the multifaceted personality traits of civil heroes, through semi-structured interviews with 50 eyewitnesses and participants in relief efforts following the Wenchuan Earthquake of China.

**Results:**

Civil heroes are ordinary individuals who display profound loyalty to the nation and its people, uphold a steadfast belief in self-reliance, boldly confront risks and challenges, continually surpass themselves, and strive to accomplish remarkable feats. The personality traits encompass five dimensions (i.e., patriotism and love for the people, independence and self-improvement, relentless striving and perseverance, selflessness and fearlessness, and grounded in facts and innovation), which intricately intertwine and collectively shape the luminous image of civil heroes, serving as a potent catalyst for social progress and civilization.

**Discussion:**

The research augments the theoretical framework and provides a more comprehensive and nuanced understanding of civil heroes, inspiring more individuals to dedicate themselves to societal welfare and foster a more harmonious and beautiful society.

## 1 Introduction

Heroes are not a monolithic entity; rather, “different people have different heroes” (Goethals and Allison, 2012). Some individuals perceive heroes as embodiments reflecting dominant societal values and behavioral norms (Wecter, 1941; Campbell, 1949; Allison and Goethals, 2016), while others view heroes as those who contribute significantly to social integration within their respective eras (Smith, 1976). In addition, some researchers have explored the “dark hero” (Bernier, 2024). Among the diverse heroic archetypes, civil heroes occupy a unique position. Traditionally, civil heroes have been regarded as civilians who knowingly risk their own lives to save others from physical harm or death (Franco et al., 2011; Riches, 2018). This portrayal emphasizes not only the shared humanity of these individuals but also the voluntary and resolute nature of their actions, even in the face of imminent danger, severe injury, disfigurement, and excruciating pain. Furthermore, it underscores the noble character of civil heroes, who are motivated solely by a desire to assist others, unburdened by the anticipation of reward or recognition. Although some may argue that the perceived grandeur of civil heroes’ deeds is unrealistic, the fact remains that in times of disaster, numerous such heroes emerge. For instance, during the Wenchuan Earthquake (Huang and Li, 2009; Ye et al., 2011; Cui et al., 2011), tens of thousands of people devoted themselves to the earthquake relief efforts. Among them, many people silently made great contributions, embodying the personality traits of civil heroes.

Undoubtedly, risking one’s own safety to save others constitutes a paramount and highly admirable aspect of civil heroes. However, to regard such rescues as the sole criterion of civil heroes appears narrow-minded, it overlooks the breadth and diversity of heroic endeavors and contributions. As positive exemplars in society, the image and deeds of civil heroes frequently transcend singular rescue operations, embracing multiple facets and levels. First of all, civil heroes can be those who silently dedicate themselves to their ordinary jobs and persevere over a long period of time, such as teachers who illuminate the future of students with the light of knowledge; doctors who tirelessly fight on the frontline against the epidemic or treat patients; and environmentalists who advocate for the protection of the ecological environment. Furthermore, the actions of civil heroes often exert profound social influence, as they prioritize the greater good over personal gains and losses, with their gaze fixed on the entire society and the future. For instance, volunteers participating in rescue efforts during major disasters not only directly assist those in distress but also convey the spiritual power of boundless love. Another example is that in 2007, Autrey, a construction worker, earned international acclaim when he courageously rescued a complete stranger from an oncoming New York subway train, with his actions serving as an inspiring exemplar of heroism for innumerable New Yorkers who were eager for such manifestations of heroism (Allison and Goethals, 2016). Heroes like Autrey are role models whose actions validate the worldviews that are most cherished within a society (Kinsella et al., 2015; Solomon et al., 2014). Consequently, the portrayal of civil heroes should be diverse and rich, potentially encompassing individuals from various ages, genders, professions, and backgrounds. Nevertheless, the personality traits of civil heroes remain unclear.

Adversity reveals true feelings, and disasters bring out the personality traits of civil heroes more vividly (Franco, 2016). What personality traits do these civil heroes exhibit? Therefore, this study selects 50 eyewitnesses and relief participants who experienced the Wenchuan Earthquake in China as interviewees. It aims to explore the personality traits of civil heroes who emerged during the Wenchuan Earthquake in China through qualitative analysis. This endeavor seeks to inspire individuals to comprehend civil heroism, foster its cultivation, and aspire to emulate heroes, thereby contributing to the harmony, security, and prosperity of mankind society.

## 2 Methods

### 2.1 Design and setting

A qualitative study involving thematic analysis was conducted. Semi-structured interviews were conducted with civil heroes. The interviews with the participants were conducted at a location and time of their preference, facilitated by a single interviewer. Interviews were conducted face-to-face by the researchers over a 22-week time scale. The reporting of the study adhered to the COREQ guidelines (Tong et al., 2007).

### 2.2 Participant recruitment and selection

Purposeful sampling is a frequently used approach in qualitative research to select information-rich cases efficiently, maximizing the use of limited resources (Patton, 2002). Research employed purposeful sampling and established inclusion and exclusion criteria (Choo et al., 2015). The establishment of these criteria enables to ensure the homogeneity and representativeness of the sample, as well as to enhance the reliability and validity of the research. The inclusion criteria consist of the following three aspects: (i) being affected by the earthquake but still actively participating in earthquake relief efforts, (ii) being involved in post-earthquake rubble search and rescue operations, and (iii) being long-term volunteers in the disaster area. In addition, the exclusion criteria are follows: (i) being unable to accurately share their relief experiences due to physical illness, (ii) being short-term volunteers (with a service duration of less than 1 day), and (iii) only providing non-on-site support such as online promotion after the disaster without actually participating in the relief work. The formulation of these criteria helps to ensure the homogeneity and representativeness of the sample, which also contributes to enhancing the reliability and validity of the research.

All researchers have been paying close attention to reports about disaster relief heroes for years and therefore are considered ‘insider’ (Bonner and Tolhurst, 2002). Recruitment and data collection were ongoing until data saturation was reached. The definition of data saturation as outlined by Fusch and Ness (2015) was adopted, where the point is reached when no additional distinct patterns or categories emerge from the collected data (Fusch and Ness, 2015).

An invitation to participate in the study was disseminated through social media platforms. Potential participants who expressed an interest were given a formal invitation, a participant information sheet, and a consent form. A week’s time was granted to potential participants during which they could contact the researchers to schedule an interview.

### 2.3 Data collection

Semi-structured interviews are an effective method for collecting data. It combines “a certain degree of control with a certain amount of freedom to develop the interview” (Wallace, 1998). Semi-structured interviews usually make use of a pre-prepared interview schedule, which would facilitate the emergence of distinct differences and similarities among respondents. And it possesses sufficient flexibility to conduct further in-depth exploration by employing suitable prompts to delve into interesting aspects or those areas where information acquisition proves challenging (Prescott, 2011).

The formulation of the interview schedule referred to McCracken’s (1988) four-step model and certain modifications were made. The first stage encompasses a detailed literature review, to establish “an inventory of the categories and relationships that the interview must investigate” (McCracken, 1988), covering aspects such as earthquake disaster (Chen et al., 2018; Dunford and Li, 2011), the impacts of earthquake (Huang and Li, 2009; Ye et al., 2011; Cui et al., 2011), and disaster relief (Moreno and Shaw, 2018; Hou and Wu, 2020). The second stage is the self-reflection stage, and it is crucial to utilize existing knowledge and experience in the early stages to see additional areas that the interview should address. However, self-reflection occurs throughout all stages of the research process, often unconsciously. Then, the third stage is to draft a preliminary outline and the questions in the outline are rather broad. The fourth stage involves conducting pre-interviews with potential interviewees. And post-interviews, a careful evaluation is carried out to determine which questions are well-received and can elicit comprehensive answers, and which are unclear or too broad in scope. Finally, the fifth stage is to specifically modify the inappropriate questions and formulate the interview schedule. For example, the overly broad question about post-disaster experiences is refined into more specific questions about how to cope with challenges. The finalized interview schedule (Table 1) is divided into three parts: life history, post-disaster experiences, and reflections. The life history segment aims to elicit narratives from the interviewees regarding their learning experiences, professional endeavors, and personal life prior to the disaster. The post-disaster experiences segment, on the other hand, endeavors to reconstruct the specific details pertaining to the interviewees’ life and work status subsequent to the disaster. The reflections section primarily invites the interviewees to evaluate their own actions and those of others, contemplating the intricate interplay between various life factors and its implications.

**Table 1:** Interview questions.

Life history	Where did you grow up?
	What is your family background?
	What is your educational experience?
	What specific jobs did you do before the earthquake?
post-disaster experiences	When did you start to participate in the earthquake relief?
	How did you get involved in the earthquake relief?
	What drove you to participate in the earthquake relief?
	What did you do specifically in the earthquake relief?
	What was your biggest concern during the earthquake relief?
	How did you solve the problems encountered during the earthquake relief?
	How were your physical and mental conditions during the earthquake relief?
reflections	How do you view your actions during the earthquake relief?
	What do you think is your biggest gain during the earthquake relief?
	What do you think is the biggest change in yourself during the earthquake relief?

The interview is conducted in an environment that is both convenient and comfortable for the interviewees, and the audio recording commences only after the researcher verbally reconfirms informed consent. The transcription is conducted word-for-word to accurately capture every utterance (Oliver et al., 2005). The researchers recorded reflections and impressions in immediate post-interview notes, facilitating contemporaneous data consideration.

### 2.4 Data analysis

Following the completion of all interviews, an initial analysis of the data was conducted, guided by the methods outlined by Braun and Clarke for thematic analysis (Braun and Clarke, 2022), and the software Nvivo12Plus was employed to code line-by-line for in-depth analysis.

The first step, ‘immersion’, encompassed gaining an understanding of the interview data through repeated listening to the audio recordings and careful review of the transcripts and field notes. The reflections and impressions of researchers following each interview were documented in field notes, which contributed to the thematic analysis.

Then, generation of initial codes involved recognizing raw data that could be classified in a meaningful way (Austin and Sutton, 2014). The raw data was decomposed into smaller, more manageable segments, such as sentences or short paragraphs. Through meticulous analysis, a variety of initial codes, including hope for national prosperity, concern about state affairs, facing difficulties bravely for the country, service to the people, putting one’s life at risk to save others, lack of pursuit of fame and wealth, fulfillment of duties, tireless work, self-rescue, continuous self-improvement, scientific rescue methods, scientific construction approaches, innovative technologies, and innovative thinking were pinpointed.

Finally, transitioning from creating codes to integrating broader themes entailed analyzing similarities with the findings of published studies, followed by discovering and collating broader themes that captured the core meaning of participants’ responses and patterns of responses (Castleberry and Nolen, 2018). Specifically include three levels: (i) a comparative analysis. A comparative analysis was conducted between the initial codes and the findings of extant published studies, aiming to discern both the congruencies and disparities between collected data and the existing body of knowledge, (ii) clustering similar code. A set of key concepts, including patriotism, altruistic service to the people, proactivity, courage to win, perseverance in continuous efforts, tenacious striving, selfless dedication, fearlessness of hardships, practicality, and innovation were extracted, and (iii) an in-depth integrative. The above concepts were synthesized into five overarching themes, e.g., patriotism and love for the people, independence and self-improvement, relentless striving and perseverance, selflessness and fearlessness, and grounded in facts and innovation.

The process was iterative, with outcomes from various stages of analysis undergoing multiple rounds of discussion and revision.

### 2.5 Validity and reliability/rigor

This paper employs “triangulation” (Thurmond, 2001) as a strategy to ensure the validity of the research. The first angle pertains to the researchers’ backgrounds, characterized by their strong research capabilities, deep theoretical foundation, and notably, all members possess postgraduate degrees with experience in qualitative work. The second angle focuses on the research methodology, specifically utilizing thematic analysis, a rigorous yet flexible approach to unraveling complex data sets. The third angle focuses on the sources of data, for which researchers meticulously selected participants from various geographical regions and diverse backgrounds to ensure that the collected data could enhance the universal applicability of the research findings. To further verify the credibility of the study, researcher collaborated with experts in the field of qualitative research to simultaneously code the same data sets, engaging in a comparative analysis of the independently derived coding outcomes to assess their consistency. The coding results reveal a high level of congruence, attesting to the robustness and reliability of the research findings (Table 2).

**Table 2:** Comparison of encoding results.

Data	Researcher coding	Expert 1 coding	Expert 2 coding
In times of crisis, everyone strives to alleviate the country’s burdens	Deep love for the country	Strong affection for the country	Heartfelt devotion to the country
On that day, we started helping set up tents for the victims, offering aid wherever needed.	Providing assistance to the victims	Serving the victims	Aiding the victims
Rather than relying on others, we saved ourselves	Self-rescue	Save oneself	Rescue oneself
Despite post-earthquake dangers, I transmitted disaster updates overnight.	Confront the challenge head-on	Meet the challenge directly	Tackle the challenge head-on
For the first two days after the earthquake, I did not sleep, focusing solely on disaster relief	Work non-stop day and night	Work around the clock	Work round the clock
It is my responsibility to persist in opening up the road	Adhere to responsibilities conscientiously	Discharge responsibilities conscientiously	Work hard and responsibly
I do not seek reward; I just want to help with earthquake relief	Selfless giving	Unselfish dedication	Wholehearted giving
In times of crisis, I stand at the forefront, unafraid of risks	Fearless giving	Unafraid to give	Fearless contributing
my colleagues and I assessed the damage, surveyed the terrain, and formulated a locally suitable plan	Proceeding from the facts	Taking facts as the starting point	Using facts as the foundation
Rebuilding is not only about constructing houses, but also about innovatively integrating the concept of sustainable development	Explore and innovate	Seek and innovate	Pioneer and innovate

### 2.6 Ethical considerations

The present work is an interview study and falls within the scope of human subject research (White, 2020). To comprehensively safeguard the rights and interests of the interviewees and ensure that they are protected from any form of harm during the interview process, this study adheres to four ethical principles: informed consent, privacy protection, harm avoidance, and fairness and rationality.

The principle of informed consent means that before the interview, a face-to-face meeting was held with interviewees to clarify the interview’s core purpose (Nijhawan et al., 2013). Their rights, such as querying and declining inappropriate questions, and obligations, like sharing relevant personal experiences, were informed. Ample time was provided for their inquiries, which were answered patiently. After their full understanding, their voluntary signature on the informed consent form was ensured, without any coercion, to safeguard their right to autonomous choice and legitimate rights and interests.

The principle of privacy protection requires that all privacy-related data in the research must be kept confidential (Arifin, 2018). During data collection, interviewees’ real-identity information is replaced with anonymous codes to prevent identity exposure. Interview records and audio transcripts are encrypted and only accessible to researchers during storage to prevent illegal acquisition or leakage. After the research, any unnecessary personal data is deleted to fully protect interviewees’ privacy.

The principle of harm avoidance states that if interviewees show negative emotions like anxiety or distress during the interview, it will be suspended at once (Dempsey et al., 2016). Then, offer psychological reassurance and adjust the questioning direction. After the interview, continuously monitor their conditions and provide necessary assistance promptly.

The principle of fairness and rationality requires the careful refinement of the interview outline to ensure that the questions are unbiased (Orb et al., 2001). During the interview, objectivity should be maintained and all interviewees should be treated equally. After the interview, the information should be objectively analyzed to accurately present their viewpoints. These four ethical principles form the cornerstone of this research, ensuring its scientific, ethical, and reliable implementation.

## 3 Findings

**Figure 1:**
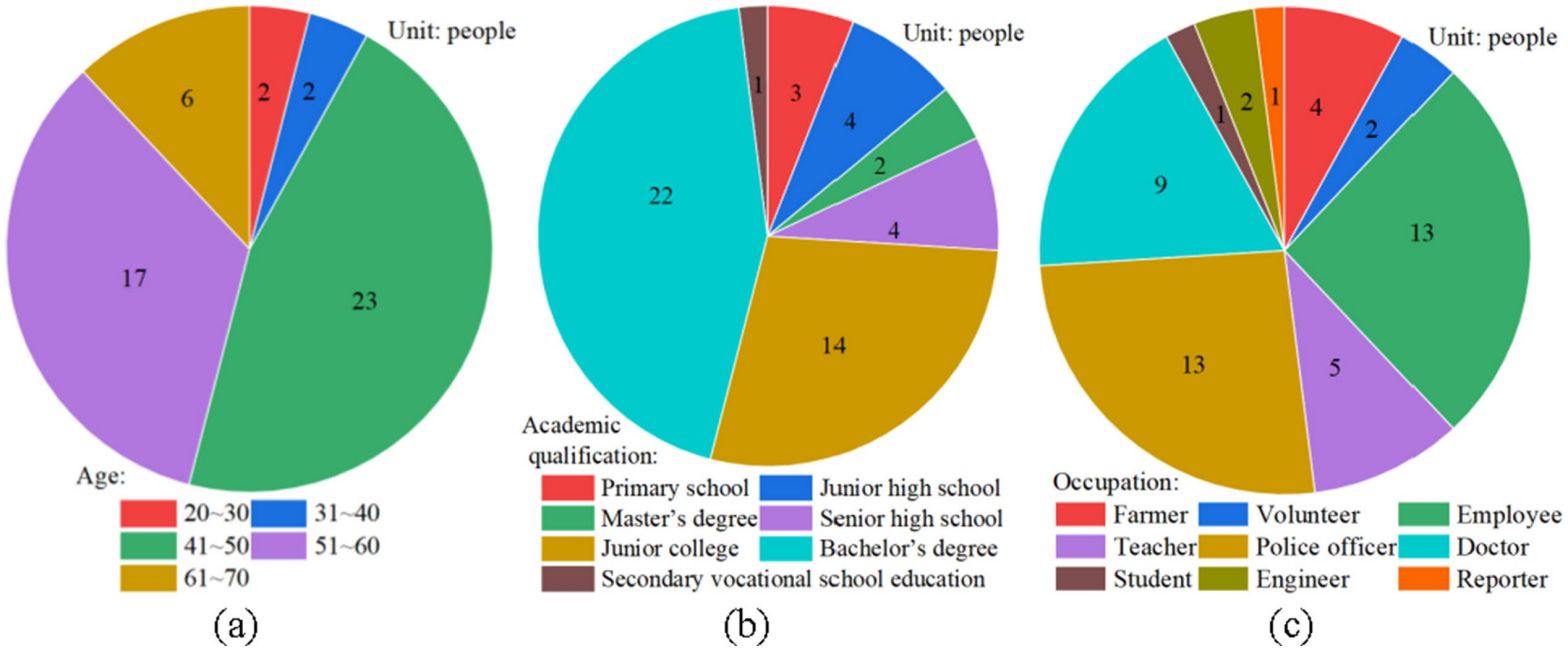
Respondent characteristics: **(a)** participants age, **(b)** participants academic qualification, and **(c)** participants occupation.

Fifty participants from various regions were interviewed, with 74% being male and 26% female, as depicted in Figure 1. The majority of the participants were aged between 40 and 60. Among them, 48% hold a bachelor’s degree or above. The participants represented a diverse range of professions, including farmers, police officers, doctors, teachers, employees, reporters, engineers, volunteers, and students, the detail of participants information can refer to Appendix Table A1. The average length of the digital audio recordings was 65 min (range: 50–90 min).

**Figure 2:**
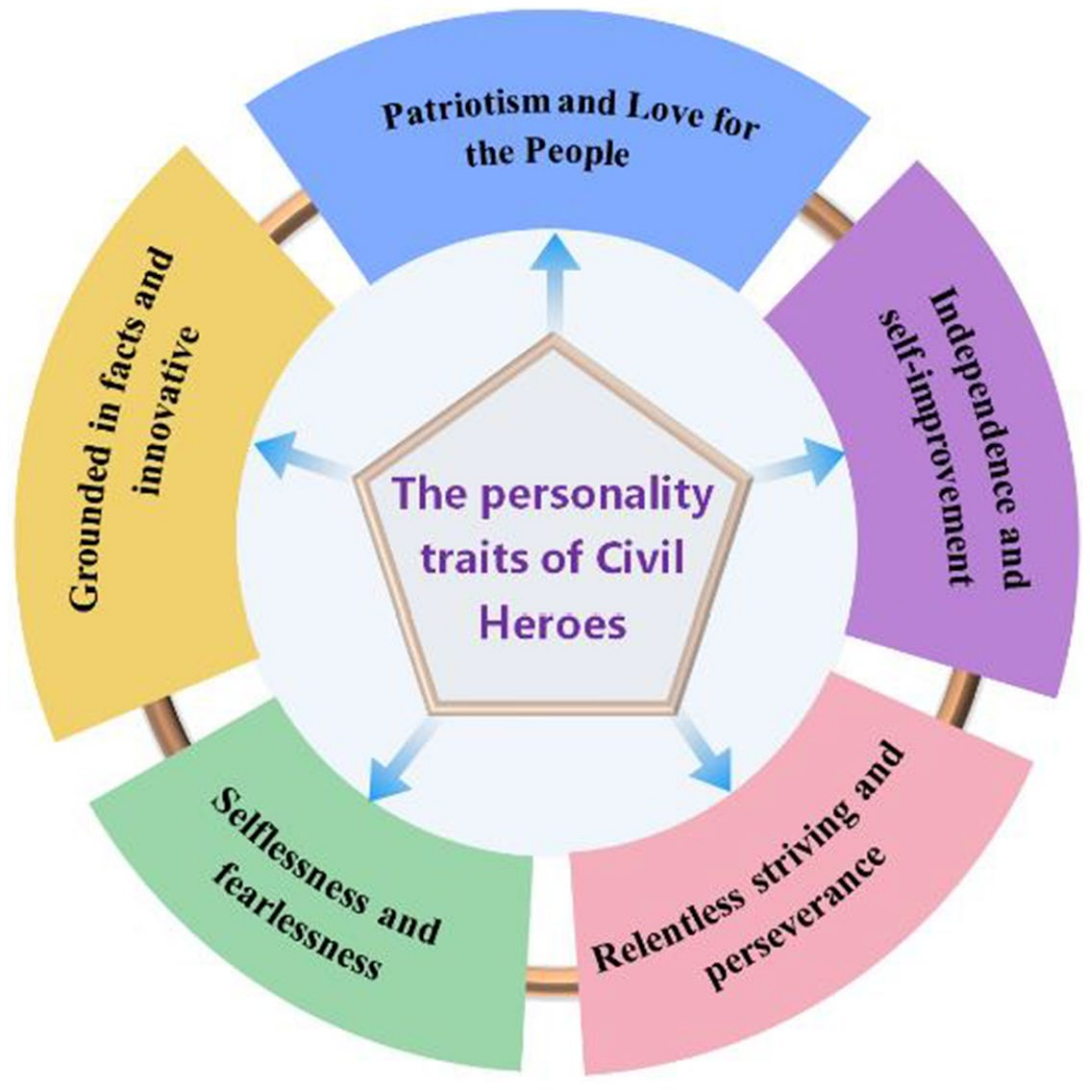
Conceptual representative of the themes.

Five themes were developed from the thoughtful analysis according to the interview data: patriotism and love for the people, independence and self-improvement, relentless striving and perseverance, selflessness and fearlessness, and grounded in facts and innovative, a conceptual representation of themes is as shown in Figure 2.

### 3.1 Patriotism and love for the people

Loving one’s country and its people is the strongest emotion (Sun, 2024), and participants hold such profound affection and deep love. After the Wenchuan Earthquake, participants devoted themselves to relief and disaster reduction, displaying deep patriotism. A police officer said, “*With so many gone in an instant, the living must shoulder responsibilities, excel in our work, and strive to be worthy of our country*” (Participant 38). An injured teacher insisted on teaching, stating, “*Teaching and educating is noble. It’s how I contribute to my country’s greatness*” (Participant 25). An employee vowed, “*Whenever the motherland calls, I’ll march forward without hesitation*” (Participant 35).

On the disaster frontline, participants prioritized the safety and well-being of the victims, offering them a variety of assistance. A construction aid remarked, “*It’s not easy for disaster victims to build a house, thus safety is paramount. Reinforcing with cement and including structural columns, ground beams, and ring beams enhances stability and safety*” (Participant 42). A farmer stressed, “*If the way had not been cleared, victims would have starved. Our homes stood, so we had food, but those whose homes fell were left hungry*” (Participant 37). Numerous participants went above and beyond to fulfill victims’ needs. For instance, Participant 31 aided in constructing a water plant during water shortages, while Participant 20 assisted victims in obtaining building materials on credit during house construction.

### 3.2 Independence and self-improvement

Many participants in the disaster area did not hesitate or rely solely on others, but instead adopted an optimistic attitude to forge ahead and create a better future in the aftermath of the earthquake. One participant recalled, “*During that time, we saved ourselves, without contemplation of relying on others. When our homes collapsed, we scavenged for materials amidst the ruins and constructed makeshift shelters. When food became scarce, we patiently cleared rubble, sowed seeds, and grew vegetables to sustain ourselves*” (Participant 1). Another participant concurred, stating, “*As long as individuals do not abandon themselves, countless possibilities lie ahead*” (Participant 20). A volunteer who traversed thousands of miles to offer aid expressed awe, remarking, “*The resilience displayed by the victims is truly remarkable! For the first time, I have witnessed the indomitable spirit of humanity, and I firmly believe that no obstacle is insurmountable*” (Participant 49).

In the earthquake relief efforts, participants faced myriad challenges but confronted them head-on with determination and responsibility. For example, with insufficient disaster relief equipment, one participant resorted to “*digging through the ruins by hand*” (Participant 27). Participant 21 recounted the experience of searching for disaster relief equipment across multiple sites and said, “*Braving the risk of aftershocks, I delivered the equipment to the frontline of rescue through the night*” (Participant 21). Some participants resolutely took unconventional actions in the extreme environment with limited communication and resources, undaunted by potential future accountability. One participant, who is a policeman, broke protocol solely to save lives, noting, “*I utilized seized motorcycle fuel to keep hospital generators running, ensuring timely surgical treatment for quake victims*” (Participant 15).

### 3.3 Relentless striving and perseverance

In response to the sudden disaster and urgent rescue demands, participants swiftly mobilized, displaying remarkable resilience and endurance. Participant 35, a medical staff member, recounted, “*In the 3 days post-earthquake, we did not have a moment’s rest, akin to battling in a war, with exhaustion etched on our faces and red-rimmed eyes*” (Participant 35). Participant 36 echoed this sentiment, emphasizing, “*Medical staff toiled day and night, snatching brief naps on benches as respite from exhaustion. Despite a grueling workload spanning over 10 days, all have lost weight yet remain steadfast, uncomplaining, and unwavering*” (Participant 36). Amidst the difficult surroundings, complaints were absent, and there were no instances of withdrawal. Participant 12 vividly recounted his endurance under the scorching sun, reeking of sweat and exhaustion, with his white shirt drenched in layers of perspiration. Similarly, Participant 9 remarked on the unusually intense sun that year, saying, “*I was so tanned that acquaintances failed to recognize me*” (Participant 9).

With exceptional responsibility, participants exemplified the depth of conscientiousness. Despite the tragic loss of loved ones in the disaster, they suppressed their grief and fully committed to relief efforts. Participant 28, whose mother passed away in the quake, remained steadfast, saying, “*Days later, my mother was found, but not by me. I was absent during her burial, busy organizing food distribution and maintaining order. I understand my life’s duty, and prioritize the collective over my personal loss*” (Participant 28). Similarly, another Participant emphasized, “*In the face of great disasters, it’s not a matter of what I should or should not do, but rather, it’s about what I must do to fulfill my responsibilities*” (Participant 38).

### 3.4 Selflessness and fearlessness

The participants, despite their ordinary backgrounds, demonstrated remarkable selflessness and fearlessness. Instead of prioritizing the safety of their loved ones, they resolutely committed themselves to the rescue efforts. For instance, Participant 8 left his family to assist in severely impacted regions, recalling, “*Leaving without a tent or provisions, I knew my wife and eight-year-old child faced hunger and rain in the mountains. Yet, I chose to prioritize aiding others over tending to my own family’s needs*” (Participant 8). These participants fought on the frontlines of earthquake relief, driven not by the pursuit of fame or wealth. As Participant 2 noted, “*In the face of catastrophes, all personal interests and distractions are set aside*” (Participant 2). Participant 32, who made multiple trips deep into the mountains to evacuate trapped victims, remarked, “*I never considered accolades or rewards; my sole purpose was to rush in and rescue the victims*” (Participant 32).

Participants fearlessly rushed toward the most dangerous places, engaging in heart-stopping contests with death. One participant, a driver, navigated the mountainous roads with vehicles loaded with supplies and recounted, “*I am on the road all day long. Sometimes, aftershocks strike, and stones roll down from the mountains, yet I keep driving. I have to be at the forefront, delivering supplies to the victims as fast as possible*” (Participant 22). Participant 6 attested, “*Many people truly charged forward to the frontline. Nobody hesitated because of the danger; everyone was eager to go*” (Participant 6). To save the lives of the victims, the participants repeatedly found themselves in perilous situations, yet they showed no signs of fear. Participant 14, who rescued victims from the ruins, narrated, “*During the rescue, shaking objects on upper floors posed a grave threat of injury or death. Yet, we fearlessly persevered in saving lives*” (Participant 14).

### 3.5 Grounded in facts and innovative

Practical and realistic approaches have been consistently implemented throughout the entire process of earthquake relief. Prior to devising strategies tailored to reality, participants first assess the situation. Taking Participant 33 as an example, he allocated machinery flexibly based on road damage and recalled, “*For severely collapsed sections, high-power equipment would be prioritized to open temporary passages, and other equipment would then follow suit to expand the working area until the site is completely cleared*” (Participant 33). Likewise, Participant 45 assisted victims in constructing satisfactory houses, emphasizing, “*It rains a lot here, so I installed a canopy outside the house. My primary concern is to meet the people’s daily needs while aligning with the local conditions*” (Participant 45).

Innovation is paramount in overcoming challenges. Participants demonstrated innovation in methods, technology, and mindset. Notably, Participant 48, despite frequent aftershocks and equipment shortages, devised an effective ‘aftershock warning system’ using a mere half-empty water bottle. Similarly, Participant 31, who introduced technology to expedite disaster recovery, remarked, “*I invited technicians to train victims, thereby establishing specialized economic zones and production hubs*” (Participant 31). Furthermore, Participant 34 advocated for ecological restoration projects, emphasizing that reconstruction efforts must address long-term development issues, fostering the harmonious integration of humanity with mountains and rivers, and ensuring the simultaneous development of culture and economy.

## 4 Discussion

### 4.1 General discussion

Fifty eyewitnesses and participants in relief efforts following the Wenchuan Earthquake were interviewed about their unique experiences. The obtained results revealed that the personality traits of civil heroes is embodied in five pivotal aspects: patriotism and love for the people, independence and self-improvement, relentless striving and perseverance, selflessness and fearlessness, as well as grounded in facts and innovative. The research underscores the importance of acknowledging that each individual possesses the potential to become a hero by contributing their unique strengths to the nation, the global community, and humanity at large. In an environment fraught with risks and challenges, it is imperative that we accord widespread attention and profound respect to civil heroes, while concurrently contemplating avenues to embody noble qualities in our own lives.

Qualitative research can more accurately capture the personality traits of civil heroes, and the conclusions drawn from it share both similarities and differences with the preliminary conception of these traits. In terms of similarities, both accounts emphasize civil heroes’ remarkable courage and responsibility in facing adversity, their prioritization of others’ or society’s well-being over personal interests, their accomplishments in their fields that contribute to societal advancement, and their origin from the ordinary populace, embodying greatness amidst the commonplace. As for the difference, firstly, the initial focus was on civil heroes’ external traits like courage, dedication, and contributions. In contrast, qualitative research revealed their core emotion: patriotism and love for the people, enriching our understanding of their multifaceted image. Secondly, the qualitative research emphatically underscored two pivotal aspects: ‘independence and self-improvement’ coupled with ‘relentless striving and perseverance.’ Although not explicitly mentioned in the initial conception, these elements are crucial in elucidating how civil heroes uphold their convictions, foster personal growth, and triumph over adversity (Goldstein and Brooks, 2012). Thirdly, adding ‘grounded in facts and innovative’ underscores heroes’ ability to stay competitive and create value in complex environments, aligning their portrayal with contemporary societal needs. Lastly, while the initial conception emphasized the ‘extensive influence’ of civil heroes, inspiring numerous individuals to emulate them (Goethals and Allison, 2012), this aspect was not as prominently featured in the qualitative research. This may stem from the latter’s concentration on the internal qualities and behavioral manifestations of civil heroes, rather than their broader societal impact. Nonetheless, both perspectives contribute valuable insights to the comprehensive understanding of civil heroes.

Several connections of the present work and the previous study on heroism can be observed, particularly in relation to established theories of prosocial behavior, altruism, and resilience in crisis situations. Prosocial behavior often involves actions that benefit others or society as a whole (Dovidio et al., 2006; Staub, 1978). The civil heroes’ dedication to helping those in need after the earthquake, which is a manifestation of their love for the people, clearly aligns with this concept. The self-initiated actions directly assist those in need and benefit the entire community; thereby demonstrating a classic example of what prosocial behavior entails. Regarding altruism, the selflessness and fearlessness of civil heroes are highly relevant. Altruism is characterized by unselfish concern for the welfare of others (Piliavin and Charng, 1990; Elster, 2006). Civil heroes who put themselves at risk without hesitation to assist others during the earthquake relief efforts, exhibit a high level of altruism. In relation to resilience, the traits of independence and self-improvement, as well as relentless striving and perseverance, are closely related. Resilience refers to the ability to bounce back from adversity and continue to function effectively (Windle, 2011; Southwick et al., 2014; Sisto et al., 2019). The civil heroes’ independence and self-improvement enable them to face the challenges of the disaster independently and continuously improve their abilities to better deal with the situation. The relentless striving and perseverance are evident in their unwavering efforts in the long-term relief work, despite the difficulties and setbacks they encounter. It demonstrates that they possess strong resilience, which is consistent with the understanding of resilience in existing theories. Overall, the study on the personality traits of civil heroes in the context of earthquake relief efforts is in line with the existing theoretical frameworks of prosocial behavior, altruism, and resilience in crisis situations, further validating and enriching the theories.

The traits of ‘grounded in facts and innovative’ exhibited by civil heroes constitutes a significant highlight of this study. Previous research on heroism has predominantly focused on aspects such as bravery, self-sacrifice, and moral excellence (Franco et al., 2011; Riches, 2018; Fagley, 2024), yet it has often lacked an in-depth exploration of the innovative behaviors demonstrated by civil heroes that are rooted in factual analysis. It confirms that when confronted with problems and challenges, civil heroes are capable of calmly analyzing actual situations and innovatively addressing difficulties, providing a new perspective for the research on heroism.

### 4.2 Strengths and limitations

The research design incorporated principles of credibility, dependability, confirmability, reflexivity, and transferability to ensure the reliability of the findings. By deeply engaging and immersing themselves in the data, the researchers enhanced the credibility of their interpretations. The study focuses on the roles and actions of individuals who participated in relief efforts following the Wenchuan Earthquake, providing valuable insights for academic research on civil heroes.

Nevertheless, it is recognized that limited resources and time have restricted role coverage, and the time elapsed since the 2008 Wenchuan Earthquake and external narratives can affect interviewees’ memories, which will undoubtedly undermine result generalizability. For the future work, more resources will be allocated and the data-collection time will be extended to cover more diverse roles, and the attention will be paid to adopting a cross-checking approach, with the information being verified through sources such as official reports and news records. Besides, it’s worth noting that cultural and historical differences across nations may impact disaster-related decision-making and behavior (Franco et al., 2011; Sun, 2024), limiting the generalizability of our findings. Future research will broaden the study of civil heroes within a cultural and historical framework, exploring similarities and differences in their portrayal and the evolution of their meanings across societies. Through comparative analyses, the aim to contribute new perspectives and theoretical frameworks to the study of civil heroes in diverse contexts.

## 5 Conclusion

The personality traits of civil heroes in the context of earthquake disasters were explored using qualitative methods. Semi-structured interviews were conducted with 50 civil heroes from diverse regions and occupations, and the software NVivo 12 Plus was employed to conduct a line-by-line coding for in-depth analysis of the gathered experiences and insights. It demonstrated that civil heroes display a variety of personality traits, including patriotism and love for the people, independence and self-improvement, relentless striving and perseverance, selflessness and fearlessness, as well as being grounded in facts and innovative, enriching the gaps in the research field of civil heroes. It contributes to the understanding of civil heroism and establishes a solid foundation for scholars to delve deeper into its nuances. Furthermore, the findings hold valuable implications for future disaster relief work. Specifically, it is crucial to recognize and harness the important role of civil heroes while strengthening the construction of professional rescue teams. By integrating these efforts, a disaster relief system can be developed that combines professional and civilian forces. Ultimately, it is hoped that the study can inspires people to recognize everyday heroism in their communities and emulate civil heroes’ brave actions, becoming heroes themselves.

## Data Availability

The original contributions presented in the study are included in the article/supplementary material, further inquiries can be directed to the corresponding author.

## Appendix A Participants information

Fifty civil heroes who participated in the relief efforts for the Wenchuan earthquake have been interviewed, and the basic information is provided in Appendix Table A1.

**Table A1:** Participants information.

Participant	Location	Gender	Age	Academic qualification	Occupation
1	Beichuan County	Female	70	Junior high school education	Farmer
2	Beichuan County	Female	43	Secondary vocational school education	Volunteer
3	Wenchuan County	Female	68	Primary school education	Farmer
4	Wenchuan County	Male	45	Primary school education	Employee
5	Beichuan County	Female	47	Bachelor’s degree	Teacher
6	Dujiangyan City	Male	45	Senior high school education	Farmer
7	Wenchuan County	Male	55	Junior college education	Police officer
8	Wenchuan County	Male	43	Junior college education	Employee
9	Wenchuan County	Female	41	Bachelor’s degree	Employee
10	Beichuan County	Male	36	Bachelor’s degree	Employee
11	Dujiangyan City	Male	50	Junior college education	Doctor
12	Dujiangyan City	Male	49	Junior college education	Police officer
13	Dujiangyan City	Female	53	Junior college education	Employee
14	Dujiangyan City	Male	48	Bachelor’s degree	Employee
15	An County	Male	29	Bachelor’s degree	Police officer
16	An County	Male	41	Junior college education	Employee
17	Shifang City	Male	45	Junior college education	Doctor
18	Shifang City	Male	56	Junior high school education	Employee
19	Shifang City	Male	28	Senior high school education	Student
20	Mianzhu City	Female	47	Junior high school education	Volunteer
21	Guangyuan City	Male	57	Bachelor’s degree	Engineer
22	Guangyuan City	Male	60	Junior college education	Employee
23	Guangyuan City	Female	60	Senior high school education	Teacher
24	Guangyuan City	Male	39	Bachelor’s degree	Employee
25	Qingchuan County	Female	51	Junior college education	Teacher
26	Qingchuan County	Male	54	Junior college education	Teacher
27	Pingwu County	Male	58	Bachelor’s degree	Police officer
28	Beichuan County	Male	45	Senior high school education	Police officer
29	Pingwu County	Female	47	Junior college education	Doctor
30	Pengzhou City	Male	51	Bachelor’s degree	Teacher
31	Pengzhou City	Male	47	Junior college education	Employee
32	Pengzhou City	Male	45	Bachelor’s degree	Police officer
33	Pengzhou City	Male	50	Bachelor’s degree	Police officer
34	Deyang City	Male	45	Junior college education	Reporter
35	Deyang City	Male	57	Bachelor’s degree	Employee
36	Deyang City	Male	57	Bachelor’s degree	Doctor
37	Songpan County	Male	70	Primary school education	Farmer
38	Xiaojin County	Male	56	Bachelor’s degree	Police officer
39	Lixian County	Female	45	Bachelor’s degree	Doctor
40	Lixian County	Male	56	Junior high school education	Driver
41	Luliang City	Male	58	Bachelor’s degree	Doctor
42	Datong City	Male	60	Junior college education	Employee
43	Zhangjiakou City	Male	49	Bachelor’s degree	Police officer
44	Shenyang City	Female	54	Master’s degree	Doctor
45	Qinhuangdao City	Female	64	Bachelor’s degree	Engineer
46	Dalian City	Male	64	Bachelor’s degree	Doctor
47	Shijiazhuang City	Male	45	Bachelor’s degree	Police officer
48	Qingdao City	Male	48	Bachelor’s degree	Police officer
49	Qingdao City	Male	46	Bachelor’s degree	Police officer
50	Jinan City	Male	70	Master’s degree	Doctor
